# Crosstalk between Oxidative Stress and Ferroptosis/Oxytosis in Ischemic Stroke: Possible Targets and Molecular Mechanisms

**DOI:** 10.1155/2021/6643382

**Published:** 2021-05-11

**Authors:** Jia-Xin Ren, Chao Li, Xiu-Li Yan, Yang Qu, Yi Yang, Zhen-Ni Guo

**Affiliations:** ^1^Stroke Center & Clinical Trial and Research Center for Stroke, Department of Neurology, The First Hospital of Jilin University, No. 1 Xinmin Street, Changchun 130021, China; ^2^China National Comprehensive Stroke Center, No. 1 Xinmin Street, Changchun 130021, China; ^3^Jilin Provincial Key Laboratory of Cerebrovascular Disease, No. 1 Xinmin Street, Changchun 130021, China

## Abstract

Oxidative stress is a key cause of ischemic stroke and an initiator of neuronal dysfunction and death, mainly through the overproduction of peroxides and the depletion of antioxidants. Ferroptosis/oxytosis is a unique, oxidative stress-induced cell death pathway characterized by lipid peroxidation and glutathione depletion. Both oxidative stress and ferroptosis/oxytosis have common molecular pathways. This review summarizes the possible targets and the mechanisms underlying the crosstalk between oxidative stress and ferroptosis/oxytosis in ischemic stroke. This knowledge might help to further understand the pathophysiology of ischemic stroke and open new perspectives for the treatment of ischemic stroke.

## 1. Introduction

Stroke is one of the leading causes of death and disability worldwide [1]. According to the 2020 American Heart Association statistics, approximately 795,000 people experience a new or recurrent stroke each year, with an average of one person having a stroke every 40 seconds in the United States [2]. Ischemic stroke accounts for 87% of all strokes [2]. This type of stroke begins with cerebral artery occlusion, which reduces blood flow to the brain, leading to insufficient blood glucose and oxygen, which causes metabolic changes, cell death, and brain damage [3]. Ischemia-damaged brain tissue can be divided in two areas: the ischemic core and the penumbra [4]. The ischemic core has minimal blood flow, with rapid and severe damage, and neuronal death is transient and necrotic [5]. The penumbra is a hypoperfused area at the periphery of the core that comprises half of the total lesion volume [6]. Neurons in the penumbra are fragile and respond to stress by releasing substances, activating signaling pathways, and undergoing complex dynamic changes [7], which allow the neurons to survive for hours or even days, until they ultimately die [8]. Oxidative stress, caused by an imbalance between oxidants and antioxidants [4], is a major initiator and propagator of neuronal dysfunction and death [9–11] and a key deleterious factor in cerebral ischemia [12]. In ischemic stroke, increased production of reactive oxygen species (ROS) in neuronal cells depletes the antioxidant system, thereby disrupting the balance between ROS production and consumption. An excess of ROS induces lipid peroxidation and oxidation of proteins, DNA, and RNA, which lead to neuronal dysfunction and death [13–15].

Ferroptosis is a type of iron-dependent, oxidative stress-induced cell death that has been shown to play an important role in ischemic stroke [16–20]. Ferroptosis is induced by erastin, RAS-selective lethal 3 (RSL3), and their related compounds, and it has been defined by Dixon et al. using pharmacological methods in 2012 [21, 22]. Different from apoptosis, necrosis, and autophagy in morphological, biochemical, and genetic terms, ferroptosis does not have characteristics such as chromatin condensation, nuclear atrophy, and cellular swelling. The distinctive morphological characteristics of ferroptosis are mitochondrial atrophy and changes in the structure of the mitochondrial cristae [21, 23, 24]. At a molecular level, ferroptosis is characterized by glutathione (GSH) depletion and lipid peroxidation, particularly oxidation of phosphatidylethanolamine (PE) containing arachidonic and adrenal acids [25]. It is noteworthy that ferroptosis is significantly similar to oxytosis, which is a distinctive oxidative stress-induced programmed cell death pathway [26]. However, after a detailed comparison of the roles of ferroptosis and oxytosis in the central nervous system, Fricker et al. suggested that ferroptosis and oxytosis should be considered for the same cell death pathway [24, 26–29].

The cellular processes and molecular machinery of cell death (and their association) in the brain of patients with ischemic stroke remain unclear [30–32]. However, it is known that, after ischemic stroke, a series of molecular events induced by oxidative stress overlap with the process of ferroptosis/oxytosis and that there are common molecular targets, such as lipid peroxidation and GSH depletion [33–35]. The widely used oxidative stress stimulant tert-butyl hydroperoxide was found to induce neuronal cell death that can be blocked by ferroptosis inhibitors, thus implying a crosstalk between the initial oxidative damage and ferroptosis [36]. Exploring the association between oxidative stress and ferroptosis/oxytosis might help to further understand the pathophysiology of ischemic stroke [27]. This review provides an overview of the key molecules involved in oxidative stress-induced peroxide production and antioxidant depletion after ischemic stroke, describes their role in ferroptosis/oxytosis, and summarizes the molecular mechanisms underlying the crosstalk between oxidative stress and ferroptosis/oxytosis.

## 2. Summary of Classic Pathways

### 2.1. Classic Pathways of Ferroptosis in Ischemic Stroke

#### 2.1.1. Free Iron Accumulation

Ferroptosis is dependent on excessive iron accumulation, which is an important component of lipid oxidation [37]. Under normal central nervous system conditions, iron is primarily bound to ferritin and neuromelanin. Iron is a crucial cofactor in the central nervous system [38], and it is involved in several important processes including oxygen transport, oxidative phosphorylation, myelin production, and neurotransmitter synthesis and metabolism [39]. Through the transferrin-transferrin receptor 1 system, iron is released into the cytoplasm after crossing the blood-brain barrier. The inactive form (Fe^3+^) is recognized by transferrin and moved into the cell by transferrin receptor 1. Subsequently, Fe^3+^ is converted to free iron (Fe^2+^) by 6-transmembrane epithelial antigen of the prostate 3. Free iron is partly transferred by ferritin and can be partly stored in the labile iron pool and is thus involved in lipid ROS production [40, 41]. Fe^2+^ is considered to be a cofactor for several metalloenzymes in oxidative reactions, such as lipoxygenase (LOX) and hypoxia-inducible factor prolyl-hydroxylase [42–44]. In this process, protein-bound iron is safe, whereas abnormal iron homeostasis and excess Fe^2+^ produce excess lipid peroxidation via Fenton reactions [40, 41]. Studies have observed increased total iron content and increased expression of iron and iron regulatory proteins in ischemic areas after permanent or transient ischemic strokes [45, 46]. Deferoxamine and iron chelators prevent free radical production and delay neuronal death [46].

#### 2.1.2. Lipid Peroxidation

Lipid peroxidation is the main consequence of ROS-mediated brain injury [4] and the key driving force of ferroptosis [23]. Lipid peroxide production occurs in three steps. First, the acyl-CoA synthetase long-chain family member 4, which exists mainly in the endoplasmic reticulum and the outer mitochondrial membrane, acts as a key regulator to catalyze the formation of arachidonic acid or other polyunsaturated fatty acids (PUFAs) to form PUFA acetyl coenzyme A [47]. Second, PE-PUFA is produced by the action of lysophosphatidylcholine acyltransferase 3. Third, under the action of iron ions, oxygen, and LOX, PE-PUFA is oxidized to PUFA hydroperoxide [25], which is eventually degraded into toxic 4-hydroxynonenal and malondialdehyde [48]. It is hypothesized that there are two main sources of lipid peroxidation: one is the endoplasmic reticulum, where PUFAs form lipid peroxides through the three above-mentioned steps, and the other is the mitochondrial ROS, which is indirectly involved [27, 49].

#### 2.1.3. GSH Consumption

Ferroptosis can be triggered by small molecules or conditions that inhibit GSH biosynthesis or the GSH-dependent antioxidant enzyme GSH peroxidase 4 (GPX4). Studies using the ferroptosis inducer erastin have shown that system X_C_^−^ plays an important role in ferroptosis [21]. System X_C_^−^ promotes the synthesis of cysteine-dependent GSH. First, the ferroptosis inducer erastin inhibits systemic X_C_^−^ and competitively inhibits cystine uptake, which leads to depletion of cysteine (a rate-limiting precursor of GSH synthesis). This subsequently leads to GSH depletion, resulting in an imbalance of cellular oxidants and antioxidants, which leads to cell death [50]. GSH depletion is a key feature of ferroptosis [37, 51, 52]. In an animal model of ischemic stroke, peptides containing selenocysteine inhibit ferroptosis by driving GPX4 expression, thus exerting a protective effect on neurons and reducing ischemic core [19, 53].

### 2.2. Classic Pathways of Oxidative Stress in Ischemic Stroke

Oxidative stress refers to the relative excess of ROS caused by excessive production of ROS and/or impaired degradation of ROS, which plays a key role in the pathological mechanism of ischemic stroke. After cerebral ischemia, a significant increase in the number of cellular calcium ions (Ca^2+^) is observed, followed by excessive accumulation of extracellular glutamate and an increased level of arachidonic acid. Further progression follows with increased ROS production and depletion of ROS scavengers, which deactivates the antioxidant system. The balance between ROS production and depletion is disrupted, ultimately leading to excessive ROS accumulation. Excess ROS leads to cellular dysfunction and cell death through lipid peroxidation and oxidation of proteins, DNA, and RNA, resulting in brain tissue damage [13–15]. Lipid peroxidation is one of the major consequences of ROS-mediated brain injury and ultimately leads to the production of conjugated diene hydroperoxides, 4-hydroxynonenal, which are toxic to neurons and white matter and can induce cell death [54]. Excess ROS production in ischemic stroke through oxidative stress activates the nuclear factor erythroid 2-related factor (Nrf2) [55], which induces the expression of Nrf2-targeted genes. Additionally, activating transcription factor 4 (ATF4) is found to be a progenitor transcription factor induced by oxidative stress in in vivo and in vitro experiments. Increased ATF4 mRNA levels and translation levels after oxidative stress make ATF4 overexpression sufficient to induce cell death [56].

## 3. Molecular Players in Peroxide Production (Table 1)

### 3.1. Glutamate

Glutamate is the most abundant neurotransmitter in the brain and one of the most abundant free amino acids. However, excessively high concentrations of extracellular glutamate are toxic to neurons [26]. Glutamate plays a key role in cerebral ischemia [57], with excessive accumulation of extracellular glutamate being a major factor contributing to neuronal cell death in the penumbra [58–60]. The reasons for an increase in extracellular glutamate include the following: (1) the concentrations of free glutamate and glutamine in the central nervous system are 5-10 mM and 2-4 mM, respectively [61]; (2) glutaminase in nerve cells converts extracellular glutamine into glutamate during cell lysis [62]; and (3) oxidative stress during ischemia shuts down the high-affinity glutamate transporters that usually remove extracellular glutamate [63] in nerve and glial cells.

Cell death pathways activated by glutamate in the nervous system include excitotoxicity and oxidative glutamate toxicity [24]. Excitatory toxicity is initiated by the activation of N-methyl-D-aspartic acid receptors [57, 64], which causes neuronal damage and oxidative stress after ischemia. However, most clinical trials assessing the effectiveness of N-methyl-D-aspartic acid receptor in patients with stroke have reported the ineffectiveness of this receptor [65–67]. In addition to the short time window for the treatment of stroke, another reason for the ineffectiveness of N-methyl-D-aspartic acid receptor inhibitors is that excitotoxicity might not be the single or main mechanism of neuronal death in stroke [24]. Oxidative glutamate toxicity is also thought to play an essential role [68].

Glutamate-induced HT22 hippocampal cell death is an established model system to study ferroptosis/oxytosis and has been widely used to clarify the mechanisms leading to cell death [69]. In glutamate-exposed HT22 hippocampal cells, oxidative glutamate toxicity does not induce nuclear fragmentation and chromatin condensation typical of apoptosis. The most evident damage caused by oxidative glutamate toxicity is mitochondrial swelling and loss of cristae. Similar phenomena have been observed in the developing nervous system [70] and ischemia models [71]. Morphological and biochemical data have revealed that oxidative glutamate toxicity also differs from the classical apoptotic pathway [72–74] in that it appears to depend on oxidative stress and ROS generation. Therefore, in 2001, Tan et al. have named oxidative glutamate toxicity as oxytosis [26].

Oxidative glutamate toxicity inhibits amino acid uptake by inhibiting the cystine/glutamate X_C_^−^ antiporter system (described below in more detail) [75]. When glutamate or other conditions deplete GSH by more than 80% for several hours, cell death might occur. As mentioned above, ferroptosis is consistent with oxytosis [76]. High concentrations of extracellular glutamate inhibit the X_C_^−^ system and induces ferroptosis [18]. The accumulation of extracellular glutamate can be a natural trigger for ferroptosis/oxytosis [51].

### 3.2. ROS Generation

ROS are by-products of oxygen metabolism and include oxygen ions, free radicals, and peroxides. ROS are highly reactive because of the presence of unpaired electrons. During oxidative stress, ROS can accumulate to toxic levels, leading to cell damage and functional impairment [77]. ROS are produced abundantly after ischemic stroke [4], and a biphasic pattern of free radical production by the pyramidal neurons in the hippocampal CA1 region of rats has been reported after transient forebrain ischemia. Biphasic ROS production can be inhibited by antioxidants and iron-chelating neuroprotectants [46]. This biphasic production of ROS has been confirmed in glutamate-exposed HT22 hippocampal cells [28]: within 0-6 hours after the addition of glutamate, the production of ROS increases linearly to approximately 10% of its maximum value; after 6 hours, ROS accumulation increases exponentially to 100-200 times that observed in untreated cells [26].

The mechanism of ROS generation is complex, which involves the interruption of mitochondrial respiratory chain oxidative phosphorylation, anaerobic glycolysis, Ca^2+^ influx, and activation of nitric oxide synthase. There are two main reasons for the production of ROS related to ferroptosis/oxytosis during ischemia: (1) Excessive intracellular ferrous ion (Fe^2+^) levels induce the production of a large number of reactive oxygen free radicals, which further attack and oxidize cell membrane lipids to trigger ferroptosis [47]. This is because the total iron content in the ischemic area increases significantly after an ischemic stroke [45], and the levels of transferrin receptor and transferrin, both of which are involved with iron metabolism, also increase [19, 46, 78–80]. Magnetic resonance imaging revealed increased iron deposition in severely hypoxic-ischemic brain tissue [81]. There are three reasons for the increase in ROS caused by excessive iron levels. First, the Haber-Weiss chemical reaction converts superoxide and hydrogen peroxide into highly reactive and toxic hydroxyl radicals [82]. Second, iron acts as a catalyst in lipid oxidation. Third, iron is an important component of the catalytic subunit of the enzyme LOX, the key target enzyme that catalyzes lipid peroxidation (described below in more detail) [83]. (2) Mitochondria are the center of ROS production and cell death, and free radicals generate superoxide anion radicals during the electron transfer step of oxidative respiration [4]. The most likely source of the late exponential burst of ROS (6 hours after glutamate exposure) is the reverse electron transfer of the flavin mononucleotide group of mitochondrial electron transfer chain complex I [84, 85]. The mitochondrial electron transport uncoupler disperses the mitochondrial membrane potential; blocks the second exponential phase of ROS generation, but not the first [85]; and prevents cell death. The same experiment has confirmed that mitochondrion-triggered ROS production is essential for erastin-induced ferroptosis [86].

### 3.3. Intracellular Calcium Ions (Ca^2+^)

The entry of Ca^2+^ into cells is a necessary step for oxidative glutamate toxicity, ultimately leading to cell death. Glutamate induces a large increase in intracellular Ca^2+^ [3, 87]. Experiments have shown that after adding glutamate, intracellular Ca^2+^ increases by 30-50 times, roughly parallel to the increase in ROS, but with a delay of 30-60 minutes. Certainly, Ca^2+^ influx and mitochondrial ROS production are tightly coupled [87]. Ruthenium red, an effective single transporter inhibitor of mitochondrial Ca^2+^ uptake, can prevent late ROS production and cell death. Therefore, mitochondrial Ca^2+^ influx is likely to be essential for maximum ROS production [85]. The mechanism underlying harmful calcium influx caused by oxidative stress during ischemia is mediated by the ORAI calcium release-activated calcium modulator 1 (ORAI1) Ca^2+^ channel and store-operated calcium entry (SOCE) [27, 88]. In the process of glutamate-induced oxidative stress, inositol triphosphate receptors are activated because of GSH depletion or other reasons, thereby depleting endoplasmic reticulum calcium stores and triggering the activation of ORAI1, which in turn activates SOCE to further increase the intracellular Ca^2+^ influx. The above-mentioned mechanism has been proven using inhibitors of corresponding molecules and gene knockout experiments [88]. In addition, Ca^2+^ influx is also blocked by soluble guanylate cyclase inhibitors [74] and stimulated by cyclic guanosine monophosphate (cGMP) [89]. Ca^2+^ entry is likely to occur through cGMP-gated Ca^2+^ channels [26]. Although the role of Ca^2+^ in ferroptosis has not received much attention [90], compounds that reduce Ca^2+^ influx, such as cobalt chloride and apomorphine, can protect erastin- and RSL3-induced ferroptosis [85]. Therefore, although Ca^2+^ cannot directly induce ferroptosis, it is coupled with ROS production and lipid peroxidation and is also affected by GSH depletion.

### 3.4. Lipid Peroxidation and LOX

In humans, the brain is the organ with the highest content of PUFAs; it is also rich in lipid peroxidation precursors [91]. The brain is highly sensitive to hypoxia-ischemia and free radical reactions [36]. Experiments using a rat ischemia model have shown that occlusion of the common carotid artery for 30 minutes and reperfusion for 1 hour lead to a significant increase in the production of ROS and the final products of lipid peroxidation [92, 93].

LOX is a very important enzyme in the pathophysiological process underlying ischemic stroke and the production of lipid peroxides leading to ferroptosis [94]. LOX binds molecular oxygen to specific positions of PUFAs and is classified as 5-LOX, 12-LOX, or 15-LOX according to the oxygen insertion position. In the central nervous system, 12-LOX predominates and produces 12- and 15-hydroxyeicosatetraenoic acids [95]. Germline deletion of *12/15-LOX* genes can reduce the infarct size after stroke [96, 97]. An increase in 12/15-LOX can be observed in neurons after ischemia [98]. In transient cerebral ischemia models, the inhibition of 12/15-LOX by baicalein can protect nerve cells from ischemia/reperfusion injury [34], and its degree of protection is similar to that of 12/15-LOX knockout mice [99]. Similarly, *LOX* gene depletion can prevent erastin-induced ferroptosis [100]. Experiments have confirmed that PE-binding protein 1 (PEBP1), the backbone protein inhibitor of protein kinases, forms a complex with 15-LOX to produce hydrogen peroxide. Therefore, PEBP1/15-LOX complex can be used as the main regulator of ferroptosis [101]. Similarly, 12/15-LOX plays an important role in oxidative glutamate toxicity and oxidative stress. Experiments with primary cultures of HT22 hippocampal neurons and the cerebral cortex have shown that glutamate activates LOX to produce 12-hydroxyeicosatetraenoic acid, and LOX inhibitors block glutamate toxicity [87]. Six hours after the addition of glutamate, the enzyme activity of 12-LOX increases significantly at the time point when the level of GSH is close to zero. This shows that GSH depletion causes LOX activation to precede the second stage of ROS production, and it is necessary for the exponential accumulation of ROS [26]. Additionally, 12-LOX metabolites can activate soluble guanylate cyclase to further produce cGMP. cGMP activates ORAI1 and SOCE to promote Ca^2+^ influx into cells [27, 74].

## 4. Molecular Players in Antioxidant Depletion (Table 1)

### 4.1. X_C_^−^, GSH, and GPX4

Another important consequence of oxidative stress in cerebral ischemia is the depletion of the antioxidant system, which is manifested primarily by the inhibition of cystine uptake by glutamate resulting in the loss of intracellular GSH [26, 102]. Lipid peroxidation due to GSH depletion is a key feature of ferroptosis [24]. Glutamate acts by inhibiting cystine deprivation at the site of the membrane cysteine/glutamate reverse exchange transporter (the X_C_^−^ system) [47, 75]. X_C_^−^ is a dimer composed of a light-chain subunit (xCT, SLC7A11) and a heavy-chain subunit (CD98hc, SLC3A2) [75]. The main function of the X_C_^−^ system is to mediate the exchange of extracellular cystine and intracellular glutamate across the cellular plasma membrane, and the newly imported cystine is used in the synthesis of GSH [59]. Reduced GSH is an essential intracellular antioxidant that serves as an important defense against oxidative stress [103] and is synthesized from glutamate, cysteine, and glycine in a two-step process via two ATP-dependent cytoplasmic enzymes: glutamate-cysteine ligase and GSH synthetase [104]. GPX4 is the only GSH peroxidase that accepts membrane phospholipid hydroperoxides as oxidation substrates [105–107] to protect biological membranes from peroxidative degradation [108]. Selenium serves as an essential key regulator of GPX4 biosynthesis [109]. In HT22 hippocampal cells, the intracellular GSH level becomes almost zero after 6 hours of glutamate addition [26], and cell death occurs when glutamate or other conditions deplete GSH beyond 80% for several hours. GSH is the cellular metabolite whose levels decrease the most during erastin-induced ferroptosis [35]. Intraperitoneal injection of TAT SelPep (a peptide containing selenocysteine) to induce GPX4 expression reduces the size of focal postischemic infarcts. Additionally, in a mouse model of ischemia, TAT SelPep can drive transcriptional responses resistant to reactive lipogenesis and cell death [19]. In summary, it is hypothesized that in cerebral ischemia, oxidative stress leads to glutamate accumulation, which inhibits the X_C_^−^ system and the import of cysteine [21]. Reduced intracellular cysteine levels lead to GSH depletion, loss of cellular antioxidant capacity, and inhibition of GPX4 [107], ultimately resulting in lipid peroxide accumulation and ferroptosis [110, 111].

## 5. Crosstalk between Oxidative Stress and Ferroptosis/Oxytosis in Ischemic Stroke (Figures 1 and 2)

### 5.1. GSH-12/15-LOX-ROS-Ca^2+^-Lipid Peroxidation-Ferroptosis

In ischemic stroke, oxidative stress leads to excessive glutamate accumulation and an increase in ROS via the Fenton chemical reaction. Glutamate inhibits X_C_^−^, leading to inactivation of GSH-depleted GPX4, which subsequently activates 12/15-LOX. Consequently, the activated 12/15-LOX induces lipid peroxidation. Additionally, LOX activates soluble guanylate cyclase and cGMP, resulting in a large increase in intracellular Ca^2+^ levels mediated through the ORAI1 and SOCE, thereby promoting maximal ROS production. Elevated 12/15-LOX stimulates mitochondrial production, further amplifying the oxidative stress caused by glutamate accumulation and GSH loss [112]. Finally, accumulation of toxic lipid peroxide products leads to ferroptosis [28, 113].

### 5.2. GSH-ATF4-Ferroptosis

ATF4 is a member of the ATF/CREB family of transcription factors [114]. Both erastin and RSL3 (ferroptosis inducers) activate leucine zipper transcription factor ATF4 proteins in primary neurons [53, 56]. Knockdown of the ATF4 homologous gene was found to protect the adult mouse brain from stroke-induced injury and disability [56]. The brains of ATF4^−/−^ mice are resistant to oxidative stress-induced cell death, and overexpression of ATF4 is sufficient to restore sensitivity to cell death induced by GSH depletion and to induce cell death on its own [115]. After GSH depletion, an increase in the mRNA levels of *ATF4* and an upregulation of translation efficiency in neurons lead to ATF4 overexpression and cell death [56]. Therefore, ATF4 is not only a stress-response protein but also a redox-regulated protein that affects the threshold of oxidative stress-induced neuronal death. Further investigation of the ATF4-mediated mechanism of cell death revealed that ATF4 activates X_C_^−^ [116] through the induction of xCT [117]. In glutamate-induced HT22 hippocampal cells, deprivation of eukaryotic translation initiation factor 2 alpha (eIF2*α*) inhibits the activation of eIF2*α* kinase general control nonderepressible 2 and the formation of eIF2*α*-GTP, greatly reducing the number of ternary translation initiation complexes, which prevents aberrant ATF4 translation. In summary, it is hypothesized that the ATF4-mediated pathway may be caused by cysteine deprivation, GSH depletion, and GCN2 activation mechanisms in the endoplasmic reticulum [118, 119], which may lead to eIF2*α* phosphorylation and formation of the ternary translation complex. These changes would promote ATF4 activation and ferroptosis through ATF4-mediated transcriptional stimulation of probable upstream ferroptotic genes [120]. Using a transcriptional repressor (actinomycin D) and a translation inhibitor (cycloheximide), ATF4 has been found to drive the expression of presumed ferroptotic genes, including Chac1 [121], Trb3 [122], Chop [123], CARS [124], and the xCT cystine antiporter [53]. Another mechanism mediated by ATF is the destruction of the Grx3/Grx4-2Fe/2S cluster complex upon GSH depletion. Thereafter, iron may be absorbed by the hypoxia-inducible factor prolyl-hydroxylases and by other enzymes that partner with iron, such as PCB1, and subsequently drive the expression of the ATF4 gene, eventually causing ferroptosis [111].

### 5.3. Keap1-Nrf2-GSH

Nrf2 is a stress-induced transcription factor that is maintained under nonstress conditions primarily by Kelch-like ECH-associated protein 1- (Keap1-) mediated degradation of the proteasome. Following oxidative stress, Keap1 degrades and dissociates from Nrf2, allowing Nrf2 to translocate into the nucleus; heterologously dimerize with proteins such as the small muscle tendon fibrosarcoma protein, which recognizes the appropriate antioxidant response element sequence [125–128]; and initiate transcription [129–131]. Many of the proteins and enzymes responsible for preventing lipid peroxidation and thereby triggering ferroptosis are Nrf2 target genes [130, 132]. *GPX4* and *SLC7A11* are two such Nrf2 target genes: activated Nrf2 protects cells from hydrogen peroxide and ferroptosis by directly upregulating the transcription of *GPX4* and *SLC7A11* [133, 134]. Notably, Nrf2 probably plays an important role in protecting brain cells from ischemic injury. Loss of Nrf2 function increases the extent of cerebral infarction and neurological deficits developing after ischemic events [135, 136]. In a mouse model of transient middle cerebral artery occlusion, Nrf2 expression was found to increase from 2 hours, peaking at 8 hours, and then declining at 24-72 hours [137]. Nrf2 levels were significantly higher in the penumbra than in the core region [138], which may be explained by higher oxidative stress in the former [137]. The Nrf2 activator tert-butylhydroquinone has been shown to enhance Nrf2 signaling activity and protect different brain cells from oxidative stress in vitro [135, 136, 139]. Thus, Nrf2 activation is induced by excessive ROS production after stroke, and Nrf2 protects the brain against ischemia/reperfusion injury primarily by inducing its target antioxidant genes to counteract excessive ROS production [135]. In summary, it is hypothesized that upon ischemic oxidative stress, Keap1 degrades and dissociates from Nrf2, allowing Nrf2 to translocate into the nucleus. Nrf2 heterodimerizes with the muscle tendon fibrosarcoma protein to recognize the appropriate antioxidant response element sequence and initiate transcription of genes such as GPX4, thereby inhibiting ferroptosis. The Keap1-Nrf2-GPX4 signaling pathway plays an important role in mediating lipid peroxidation and ferroptosis [130]. In addition, many of the proteins involved in the Nrf2 signaling pathway are also direct targets of lipid oxidation, and 4-hydroxynonenal has been shown to bind to the negative regulator of Nrf2, Keap1, to activate the expression of Nrf2 target genes [140]. Nrf2 also plays a role in preventing the formation of reactive lipid intermediates.

### 5.4. Clinical Studies

Although there is no complete cellular, animal, or clinical evidence for the possible crosstalk mechanism postulated above, there are studies that confirm the mechanism as a possible target for clinical treatment and encourage further exploration. First, edaravone, a free radical scavenger that has been clinically approved for the treatment of acute ischemic stroke, resists ferroptosis induced by various conditions, particularly under cystine deprivation. Similarly, edaravone inhibits ferroptosis induced in cells by the use of xCT or GPX4 inhibitors. It was confirmed that edaravone inhibits the metabolic features normally observed in ferroptosis, which concludes Fe^2+^ accumulation and increased lipid peroxidation [141]. Second, taraxasterol protects hippocampal neurons from damage due to oxygen glucose deprivation by activating the Nrf2 signaling pathway. Taraxasterol ameliorates the decrease in cell viability of hippocampal neurons induced by oxygen glucose deprivation. Notably, by inducing Nrf2 nuclear accumulation and GPX4 expression, taraxerol significantly inhibits ROS and malondialdehyde production in hippocampal neurons induced by oxyglucose deprivation/reperfusion. Therefore, it can be hypothesized that taraxerol protects hippocampal neurons from oxidative stress and ferroptosis by regulating the Nrf2 signaling pathway in ischemia [52]. Third, the Nrf2 activator octreotide protects the brain from cerebral ischemic injury by activating the Nrf2/ARE pathway [142].

### 5.5. Research and Clinical Implications

There are several implications of the crosstalk between oxidative stress and ferroptosis/oxytosis. First, salvage of the penumbra is an important target for stroke therapy [24]. Although the mechanisms of cell death in the penumbra are diverse and complex [31], the potential targets and molecular mechanisms of ferroptosis/oxytosis summarized herein provide new insights into the exploration of stroke therapeutics. Second, the study of oxidative stress has been plagued by the use of excessive micromolar-to-millimolar concentration peroxide models, which cannot be fully realized in intact cells [143]. Thus, the massive depletion of endogenous antioxidants in ferroptosis/oxytosis models provides new ideas for studying the mechanisms of oxidative stress [111]. Third, it is only in the last decade that ferroptosis has been gradually recognized and explored for its role in neurological diseases [21]. Most studies on ferroptosis have focused on experimental models induced by various chemical inhibitors. More basic experiments, including ferroptosis-related gene knockouts and cellular and animal experiments, are still needed to fully validate and explore these potential targets and molecular mechanisms. The association between oxidative stress and the molecules involved in the development of ferroptosis is still to be explored in greater depth to provide more convincing conclusions with a causal association. In addition, this review focuses on the analysis of oxidative stress-induced ferroptosis/oxytosis. Moreover, it was also found that the molecules involved in ferroptosis/oxytosis amplify oxidative stress [26, 144]; therefore, whether and how ferroptosis/oxytosis contributes further to oxidative stress need to be further explored.

## 6. Conclusions

Oxidative stress is an established mediator of neuronal loss in cerebral ischemia [145] and an initiator and propagator of neuronal dysfunction and death [9–11], which are key causative factors of cerebral ischemia [12]. Ferroptosis/oxytosis is a unique, oxidative stress-induced cell death pathway that has expanded our understanding of the role of oxidative stress in ischemic stroke [111]. Starting off from the excessive production of peroxides and the depletion of antioxidants in neurons following oxidative stress, we described the triggers of ferroptosis/oxytosis: glutamate and its oxidative toxicity, ROS generation, increased intracellular calcium levels, lipid peroxidation, and GSH depletion. Next, we provided a summary of the interactions between these molecules targets and how they amplify oxidative stress and lead to ferroptosis/oxytosis. Exploring the crosstalk between possible targets and molecular mechanisms shared by oxidative stress and ferroptosis/oxytosis has important theoretical and clinical implications for ischemic stroke pathology. This knowledge might open new perspectives for the treatment of ischemic stroke.

## Figures and Tables

**Figure 1:**
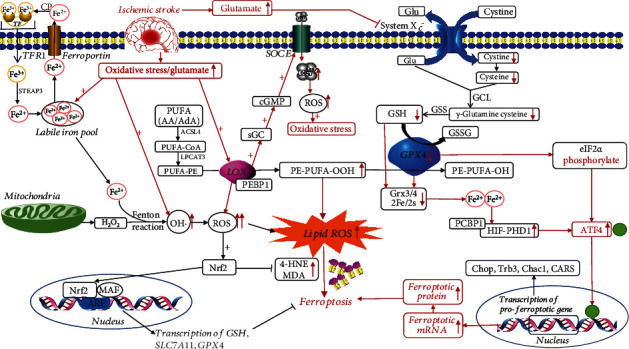
Possible targets and molecular mechanisms of the crosstalk between oxidative stress and ferroptosis/oxytosis in ischemic stroke. Red boxes and spikes indicate potential mechanisms after ischemic stroke, with upward spikes indicating an increase and downward spikes indicating a decrease. *Abbreviations*: AA: arachidonic acid; AdA: adrenic acid; ACSL4: acyl-CoA synthetase long-chain family member 4; ARE: antioxidant response element; CP: ceruloplasmin; GCL: glutamate cysteine ligase; Glu: glutamate; GSS: glutathione synthetase; GSSG: oxidized GSH; HIF-PHD 1: hypoxia-inducible factor prolyl-hydroxylase 1; H_2_O_2_: hydrogen peroxide; 4-HNE: 4-hydroxynonenal; LPCAT3: lysophosphatidylcholine acyltransferase 3; MAF: muscle tendon fibrosarcoma protein; MDA: malondialdehyde; OH·: hydroxyl radical; sGC: soluble guanylate cyclase; STEAP3: six-transmembrane epithelial antigen of the prostate 3.

**Figure 2:**
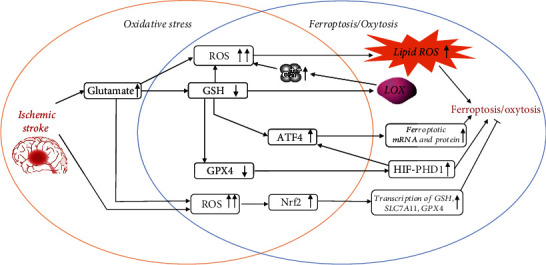
Highlight of the potential crosstalk between ferroptosis and oxidative stress. The orange circle indicates the classical pathways of oxidative stress in the process of ischemic stroke. The blue circle indicates the classical pathways of ferroptosis in the process of ischemic stroke. The intersection of the two circles indicates the potential crosstalk between ferroptosis and oxidative stress.

**Table 1:** Summary of molecular targets.

Molecular targets		Changes in ischemic stroke	Role in crosstalk between oxidative stress and ferroptosis/oxytosis	Clinical implications	References
Molecular players in peroxide production	Glutamate	Extracellular glutamate accumulation	A natural trigger which inhibits the cystine/glutamate X_C_^−^ antiporter system and promotes oxidative stress and ROS production	Glutamate-induced HT22 hippocampal cell death is an established model system to study ferroptosis/oxytosis	[18, 24, 26, 51, 57, 69, 75]
	Fe^2+^	Excessive intracellular Fe^2+^	Fe^2+^ induces the increase of ROS by three ways: the Haber-Weiss chemical reaction, catalyzing lipid peroxidation, and important component of the catalytic subunit of LOX	Iron chelators deferoxamine can prevent ROS production and delay neuronal death	[38, 39, 46, 47, 82, 83, 146]
	ROS generation	Excessive ROS generation	The key molecular which leads to the production of lipid peroxidation	The target of antioxidants	[4, 26, 28, 77, 82, 84–86]
	Ca^2+^	Intracellular Ca^2+^ increase	Ca^2+^ is associated with ROS production and lipid peroxidation	Compounds that reduce Ca^2+^ influx can protect cell erastin-induced ferroptosis	[3, 27, 85, 87, 88, 90]
	Lipid peroxidation and LOX	Significant increase of lipid peroxidation	Lipid peroxidation is the main consequence of ROS-mediated brain injury and the key driving force of ferroptosis. LOX is a very important enzyme in the production of lipid peroxides	LOX inhibitors block glutamate toxicity and reduce neuronal ferroptosis and infarct size	[17, 23, 92, 93, 96, 97, 99]
	ATF4	ATF4 overexpression	ATF4, as a predecessor transcription factor of oxidative stress in neurons, drives the expression of presumed ferroptotic genes, including Chac1, Trb3, Chop, CARS, and the xCT cystine antiporter	ATF4 knockdown protects adult rats from stroke-induced injury	[53, 56, 120–124]

Molecular players in antioxidant depletion	X_C_^−^, GSH, GPX4	GPX4 and X_C_^−^ inhibition, GSH depletion	Ultimately resulting in lipid peroxide accumulation and ferroptosis	TAT SelPep (a peptide containing selenocysteine) inducing GPX4 expression reduces the size of focal postischemic infarcts	[19, 26, 35, 102, 103, 105–107]
	Nrf2	Nrf2 activation	Nrf2 induces the transcription of proteins and enzymes, which are responsible for preventing lipid peroxidation and ferroptosis	Taraxasterol protects hippocampal neurons from damage due to oxygen glucose deprivation by activating the Nrf2 signaling pathway	[52, 130–132, 135–137, 139]

## Abbreviations

cGMP: Cyclic guanosine monophosphate
GPX4: Glutathione peroxidase-4
GSH: Glutathione
12/15-LOX: 12/15-Lipoxygenases
PEs: Phosphatidylethanolamines
PHDs: Prolyl-hydroxylases
PUFA: Polyunsaturated fatty acid
ROS: Reactive oxygen species
TF: Transferrin
TFR1: Transferrin receptor 1.
